# Factors Influencing the Response to Infectious Diseases: Focusing on the Case of SARS and MERS in South Korea

**DOI:** 10.3390/ijerph16081432

**Published:** 2019-04-22

**Authors:** Kyu-Myoung Lee, Kyujin Jung

**Affiliations:** 1 Department of Public Administration, Korea University, Seoul 02841, Korea; joanna528@korea.ac.kr; 2 Department of Public Administration and the Graduate School of Governance, Sungkyunkwan University, Seoul 02841, Korea

**Keywords:** infectious diseases, meta-analyses, severe acute respiratory syndrome (SARS), Middle East Respiratory Syndrome (MERS), South Korea

## Abstract

Following the 2003 the severe acute respiratory syndrome (SARS) and the 2015 Middle East Respiratory Syndrome (MERS) outbreak in South Korea, this research aims to explore and examine the factors influencing the response to infectious diseases, which encompasses both communicable and non-communicable diseases. Through a qualitative research method, this research categorizes the factors as inputs, processes and outputs and applies them into the 2003 SARS and MERS outbreak in South Korea. As the results conducted meta-analyses to comprehensively analyze the correlations of factors influencing disaster response from a Korean context, the findings show that the legislative factor had direct and indirect influence on the overall process of infectious disease response and that Leadership of the central government, establishment of an intergovernmental response system, the need for communication, information sharing and disclosure and onsite response were identified as key factors influencing effective infectious disease response.

## 1. Introduction

Recently, a wide array of disasters, including earthquakes, forest fires, various infectious disease outbreaks and marine accidents have occurred in Korea. Accordingly, the importance of disaster response has drawn more attention than it ever has in Korea, which was once considered a safe zone from various disasters. However, following the recent and sudden increase in the frequency of disasters, sufficient learning and legislature, as well as policy-making processes in Korea have exposed limitations in the disaster management system, relative to other countries and regions where disasters have occurred more frequently. The recent efforts to change the disaster response system after certain disasters has exposed many limitations. Under such circumstances, it is difficult to respond appropriately to disasters and there are limitations to avoiding or reducing social and economic losses caused by disasters.

After the sinking of MV Sewol, also called the Sewol ferry disaster, in 16 April 2014, the Korea Coast Guard was dissolved and the Ministry of Public Safety and Security (MPSS) took on the role of a control tower. Every episode of failed disaster response was always blamed on the lack of a control tower. Therefore, the introduction of MPSS was expected to be a panacea for effective disaster response. However, the initial response still failed during subsequent disasters, including in the Middle East respiratory syndrome (MERS) outbreak, the Geyongju earthquake and various marine accidents, which confirmed that the disaster response was still not being effectively executed. When President Moon took office, the new government underwent reorganization and in July 2017, MPSS was integrated into and absorbed by the Ministry of Interior and Safety. In 2018, the Department of Disaster and Safety Management within the Ministry of Interior and Safety took over the responsibilities that used to belong to MPSS.

Despite the persisting limitations in disaster response and management, studies related to disaster response continued to discuss the need to achieve the common goal of effective disaster management within a network with participation from regional government, central government, private organizations, citizen’s groups and military units based on independence and autonomy. [1,2,3,4,5,6,7,8,9,10,11]. Moreover, other studies outlined factors influencing disaster management and response from a macroscopic perspective and analyzed disaster management and response through awareness surveys [8,12,13,14]. Discussions on disaster resilience have been increasing in recent years. The term “resilience” refers to a concept that demonstrates the attributes of the ecosystem as a system, which has become a social science concept [15] and is being applied in disaster management. After a disaster, our society requires resilience to return to normalcy following the disaster’s physical and social impact. This can be regarded as part of the recovery phase of disaster management. Resilience includes not only a physical system but also disaster resilience at the organizational level, which is the ability to cooperate in coordinating key resources by going above and beyond for the community and minimizing operational confusion [16]. Despite the various disaster-related studies in Korea and abroad mentioned above, studies about disasters within the context of Korea, where discussions on disasters have only recently begun, have been qualitatively and quantitatively insufficient. Therefore, in addition to the existing studies, diverse and comprehensive discussions consisting of identification and securement of resources for disaster management; information sharing; timely and accurate communication and coordination with relevant agencies; networking processes that organically link disaster prevention and preparedness during non-disaster times; response during a disaster; recovery after a disaster; and learning from the experience of the disaster; are needed for effective disaster response. However, as the present study focused on disaster response, its discussion section did not cover recovery that includes resilience.

Moreover, the present study focused on infectious diseases. Despite not being the first infectious disease outbreak in Korea, responses to the severe acute respiratory syndrome (SARS) outbreak in 2003 and the Middle East respiratory syndrome (MERS) outbreak in 2015 had completely different outcomes. In 2003, upon identification of the outbreak of an emerging infection known as SARS in China, immediate nationwide preventive measures and an organic cooperation system involving the central government, health centers and quarantine stations nationwide were implemented. In the end, approximately over 8000 people worldwide, including in China, were infected and approximately 770 people died. However, Korea had only three confirmed SARS patients and no deaths; as a result, Korea was assessed as a model nation for SARS prevention by the World Health Organization (WHO). By 2015, however, more than 10 years later, Korea’s reputation regarding infectious disease response had been tarnished by systematic problems and the absence of disaster management and communication capacity. After the MERS outbreak, everyone felt the need for an in-depth examination of issues concerning how the infectious disease response system in Korea had changed over the past 10 years; the role of the central government, experts and the onsite command system and organizations at regional level; and how to solve some of those problems.

Despite the growing interest in and the need for further research, there are only few studies on the factors that determine the success and failure of infectious disease response and the responses to administrative and policy aspects. Therefore, the present study aimed to conduct a systematic investigation into the factors that influence disaster response, focusing specifically on infectious diseases by examining cases of outbreaks of infectious diseases that have occurred in Korea. The study aimed to analyze the correlations of the factors that influence infectious diseases by conducting a review of articles, precedent studies and other publications.

## 2. Theoretical and Institutional Background

### 2.1. Infectious Diseases

The present study conducted an in-depth review of the concept of infectious disease and infectious disease response, which are the subjects of its theoretical review. In the past, the terms “contagious” or “communicable disease” were generally used. However, because the term “communicable diseases” implied diseases that were transmitted from one person to another, which further implied difficulties in controlling them, the term was changed to “infectious diseases,” which encompasses both communicable and non-communicable diseases. For effective prevention and management of infectious diseases, the existing “Parasitic Disease Prevention Act” and “Communicable Disease Prevention Act” were merged. According to the “Infectious Disease Control and Prevention Act,” infectious diseases include Class 1–5 of infectious diseases, designated infectious diseases, WHO-monitored infectious diseases, bioterror infectious diseases, sexually transmitted diseases, zoonotic infectious diseases and healthcare-associated infections.

Korea has experienced outbreaks of diseases that were traditionally regarded as “diseases that occur in developing countries,” such as hepatitis A, tuberculosis, chicken pox and malaria, while cholera patients were identified for the first time in 15 years in 2016. Moreover, despite continued outbreaks of emerging infectious diseases since the SARS outbreak, there have been no noticeable changes in prevention and response measures. With the subsequent occurrence of the MERS and Zika virus in Korea and the re-emergence of cholera, an infectious disease that had not been experienced for a while, public anxiety about health safety is growing. According to the Infectious Diseases Surveillance Yearbook published by the Korea Center for Disease Control and Prevention (KCDC), the level of imported infectious diseases has been increasing every year, with 300–400 newly reported cases every year since 2010.

Experts have warned that this is only the beginning of the war against infectious diseases, due to the following reasons. With the changing global environment and increased human migration, prevention of emergence or re-emergence of infectious diseases is fundamentally impossible. Moreover, infectious diseases tend to evolve along with advances in medical technology and emerging infectious diseases are difficult to handle since they spread quickly and have no readily available treatment. In particular, knowledge about the characteristics, route of infection and control measures of emerging infectious diseases are lacking or uncertain and it is difficult to predict when, where and how these diseases may emerge. Moreover, globalization has brought with it an increased level of international trade and travel. Therefore, Korea, which has a high foreign trade dependency, is constantly exposed to the risk of imported infectious diseases.

Despite the growing anxiety and concerns about infectious diseases, studies on infectious disease response and control are lacking. After the MERS outbreak, numerous studies on the response to this outbreak were conducted [17,18,19,20,21,22,23,24,25,26,27,28,29,30,31,32,33,34,35,36,37]. However, most of the studies focused mostly on medicine and communication, with relatively fewer studies focusing on administrative fields [38,39,40,41,42,43,44,45,46]. Studies in the medical field must precede the response to infectious diseases, so that information and knowledge about the infectious disease can be applied in response measures. However, if the national infectious disease response system is not ready when an actual infectious disease outbreak occurs, then medical determination and response, as well as crisis management and communication cannot be executed properly. This is because medical response, crisis management and communication are sub-elements in such a national-scale system. Therefore, it is important to conduct studies on infectious diseases and responses in every specialty. However, there is also need for comprehensive discussions that include the establishment of laws; regulations; resources; information on infectious disease response from administrative and policy perspectives; information sharing system; and the establishment of an international cooperation system and national response system involving the central government, the regional government, private organizations and the public for effective response when an actual infectious disease outbreak occurs.

In addition to infectious diseases being difficult to handle, the MERS outbreak in 2015 also revealed that even if prevention and response measures are in place, a failed initial response can lead to an unanticipated increase in the rate and scope of infection transmission. Moreover, disaster responses do not always pan out as planned and uncertainties and complexities that emerge after the disaster must be handled. Therefore, it is necessary to identify government-level responses and make effort to improve the response system. However, previous studies on responses to infectious diseases are still lacking despite the importance of this issue. Accordingly, the present study was conducted with the consciousness of the need to analyze response systems based on past response experiences in order to effectively respond to future infectious diseases, which are a threat. The present study analyzed two cases of infectious disease outbreaks based on the factors that influence response to disaster, as identified through existing studies and theories and aimed to derive factors that have a strong influence on the effectiveness of actual disaster response.

In the following section, the factors that influence disaster response will be categorized from a system theory perspective to form a categorization framework.

### 2.2. Factors That Influence Disaster Response

Factors that influence disaster response have been identified through numerous studies over several years. Most of the studies on disaster response analyzed actual cases by applying analytical tools based on major variables presented in existing studies and theories [14,47,48,49,50,51,52,53,54] or they analyzed the factors that influence disaster response by administering questionnaire surveys to members of agencies associated with disaster response [12,55,56]. Therefore, instead of comprehensively examining the factors influencing disaster response, these studies handled the subject at a macroscopic level, focusing on the major variables. The present study aimed to organize factors and variables that influence the entire process of disaster response from a comprehensive and systematic perspective and categorize these factors and variables based on a system theory perspective in order to present an analytical framework. This concept and context are similar to those of Perry [57], who claimed that influencing factors of disaster response that are derived without classifying according to disaster types do not need different analytical frameworks since they can be described and analyzed according to the factors presented and they only differ in intensity according to the type of disaster.

The present study reviewed existing studies, focusing on those with “disaster response” and “effective disaster response” as the outcome variables. The factors that influence disaster response can be broadly categorized into financial resources, human resources, physical resources, information, education and training, leadership, intergovernmental relationships, onsite response, information sharing, environmental context, characteristics of disaster and the legal/institutional environment.

Resources that influence disaster response can be categorized into financial, human and physical resources.

Financial resources include the government’s budget for disaster response, funding to support processes involved in disaster response and support from the government or community [50,51,52,53]. Therefore, financial resources can be regarded as the disaster-related budget, the disaster management fund and financial support measures for processes entailed in Korea’s disaster response system.

Human resources included disaster response-related organizations and agencies, education and training of relevant organizations and the general public and utilization of specialists. Existing studies have pointed out that the establishment of disaster response-related organizations or crisis management centers and the securement of specialists have a very significant influence on disaster response [49,51,52,55,58,59]. This is because identification of disaster response-related organizations and agencies must come first to allow effective communication about disaster response and secure accountability in disaster response [55]. Moreover, education and training for disaster response organizations and their members has been mentioned as an element that allows effective disaster response [49,51]. Lastly, physical resources refer to securement of disaster management-related resources and establishment of disaster management facilities. As indicated in the study by Lindell et al. [51], securement of disaster response and management resources within the organization allows timely and accurate disaster response, which was expressed as disaster response equipment in the study by Jung [56]. Such physical resources can be viewed as disaster management resources and facilities and whether the expansion of negative-pressure units and emergency isolation units have occurred also influences the response to infectious diseases.

Other influencing factors besides resource-related factors include education and training. Knowledge can be explained as a collection of disaster-related information and information sharing in advance, where information about different types of disasters must be collected before the occurrence of a disaster. Factors associated with disaster information have a positive effect on disaster response, as indicated in the study by Kim [60], which reported that when a disaster information system is established the recognition of the importance of information quality and higher information quality had a positive effect on achieving disaster management duties.

Next, leadership, intergovernmental relationships and communication, onsite response and information sharing have been identified as factors that influence disaster response. First, leadership in the context of this paper refers to leadership in the central government, which can be divided into leadership from the President and leadership from central organizations and agencies. The President’s level of interest in disaster management and response and the governance style in running an organization, were analyzed as factors that have significant influence on disaster response [61]. Moreover, onsite leadership by the heads of organizations and agencies must be demonstrated in a timely manner, especially in initial disaster response, in order to prevent the disaster from spreading [50,53,56,59,62]. Second, intergovernmental relationships and communication are also factors that influence effective disaster response [50,52,53,63,64]. In addition, network was also pointed out as a factor that influences disaster response. Existing studies tended to use the concepts of network and inter-organizational cooperation without clearly differentiating them but both network and inter-organizational cooperation were analyzed as factors that positively influence disaster response [54,63,64]. One of the reasons inter-organizational communication and coordination, network and cooperation have been identified as important influencing factors is that appropriate allocation and utilization of disaster response-related resources are essential for effective disaster response. Accordingly, the present study analyzed intergovernmental relationships in order to comprehensively examine intergovernmental and inter-organizational relationships, communication and cooperation.

Information sharing has also been identified as an important factor in disaster response. Information sharing between organizations and with the general public after a disaster was found to have a positive influence on actual disaster response. In particular, a study by Hyun [6] found that information disclosure-related legislation, the organization’s budget, personal awareness and attitude and information quality influenced the effectiveness of disaster management. Among various factors influencing disaster management and response, factors associated with disaster-related information disclosure and sharing were tested for their influence on the effectiveness of disaster management. The results showed that greater information quality in information disclosure and sharing and greater personal awareness and attitude positively influenced the effectiveness of disaster management. Effective disaster response may comprise sub-variables from its outcome aspect. In a study by Denise [65], the effectiveness of disaster response was determined by measuring life loss, property damage, satisfaction of stakeholders, society’s resilience, operational efficiency and budget maximization. Moreover, a study by Byun (2014) examined fire service organizations and thus effectiveness was determined by evaluating fire containment and rescue, while efficiency was represented by reduction in damage and cost-effectiveness.

Other factors influencing disaster response include environmental factors [55,56,58,59], disaster characteristics [51,55,59] and legislative factors [3,51,55,66].

### 2.3. Introduction of Cases

#### 2.3.1. SARS

SARS stands for Severe Acute Respiratory Syndrome. It has a latency period of 10 days, after which the victim experiences high fever (≥38 ℃), coughing and respiratory distress. It is transmitted by respiratory routes to medical staff and family members who come in close contact with the patient. Complete cure is possible if treatment is administered early, where approximately 90% of infected patients recover easily within one week. However, SARS may rapidly become severe for elderly or frail patients or for patients with chronic illness, yielding a mortality rate of approximately 3.5%.

SARS became known worldwide on 11 February 2003, when the Chinese health authority announced that 305 patients with SARS had been identified in China between November 16, 2002 and 9 February 2003, five of whom had died. WHO, which had strengthened its surveillance activities in the Asian region after identifying the likelihood of the emergence of influenza, issued a worldwide warning on 12 March 2003. According to the official statistics released by WHO in November 2003, between November 2002 and July 2003, a total of 8098 suspected SARS cases from a total of 28 countries were identified and a total of 774 SARS related deaths were reported. Consequently, SARS emerged as the first new disease in the 21st century that was highly contagious and severe and its transmission through international air travel received special attention.

Since February 2003, when SARS became known for the first time, Korea continued to monitor SARS outbreak trends through WHO data and recognized the need for national quarantine measures. Accordingly, guidelines for strengthening nationwide SARS quarantine measures were passed down on 12 February 2003 and on March 16 the Korean government issued a SARS alert, in keeping with the announcement of the global SARS alert by WHO and established a quarantine system. With NIH playing a central role, all healthcare institutions, including 12 national quarantine stations and 242 health centers, maintained a 24-hour emergency operation-ready status as part of the emergency SARS quarantine measures. Moreover, a quarantine system was established by designating 41 hospitals as isolation treatment hospitals according to regions. In addition, the policy of measuring people’s body temperature as they entered the country through airports and sea ports was implemented in order to detect SARS inflow from overseas and to prevent the disease from spreading. Furthermore, follow-up investigations were conducted on people who entered Korea from SARS-risk regions and a system through which patients suspected of being infected could be transported immediately for isolation and treatment was put in place. Moreover, additional isolation measures were implemented for people who came in contact with infected patients to prevent the disease from spreading further, in addition to preventing the import of SARS. Recommendations were made to refrain from traveling to high-risk regions to further prevent the import of SARS into Korea and to encourage precautions during travel.

During the response process to the outbreak, a meeting for city and provincial quarantine officials and experts was held on 3 April 2003. On 23 April 2003, a discussion was held on measures of blocking the importation of SARS and preventing its spread. The decision to implement government-wide response measures by setting up a central SARS measures headquarter, led by the Minister of Health and Welfare and satellite stations at city and province levels was made. Subsequently on 28 April 2003, a government-wide comprehensive SARS situation room was introduced and Prime Minister Kun Goh released a general public statement, urging active cooperation from the general public regarding the response measures taken by the government. Eventually, WHO declared on 17 June 2003 that Korea had won the war against SARS, effectively subduing SARS in less than 100 days after the global SARS alert was issued [67].

#### 2.3.2. MERS

MERS stands for Middle East respiratory syndrome. The outbreak of MERS coronavirus started on 24 April 2015 following its introduction into Korea by a 68-year-old male, who worked in floriculture and was returning home to Korea after a visit to the Middle East. The patient was treated at a clinic for a fever he developed seven days after arriving in Korea but his condition did not improve. After his visit to the clinic, he received inpatient treatment for three days at Pyeongtaek St. Mary’s Hospital and was subsequently discharged. Because of continued symptoms of high fever and respiratory distress, he visited another clinic and was eventually admitted to a single-bed unit at the Samsung Medical Center in Seoul on 18 May 2015. The staff at Samsung Medical Center had learned that the patient had visited the Middle East and based on a suspicion of MERS, the doctor in charge requested KCDC to perform further testing on the patient the following day (19 May 2015). The diagnostic test performed by NIH detected Middle East Respiratory Syndrome CoronaVirus: (MERS-CoV) genes in the patient. Following the announcement of these findings on 20 May 2015, identification of the first MERS patient in Korea was officially reported [68].

**Figure 1:**
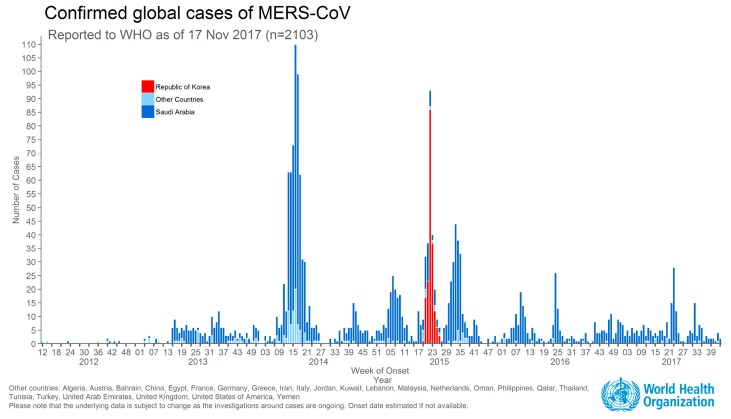
Confirmed global cases of MERS-CoV (source: https://www.who.int/emergencies/mers-cov/en/).

As shown in Figure 1, the first MERS patient in Korea visited multiple clinics and large hospitals for treatment over a 10-day period since the symptoms first appeared, during which time he came in contact with family members, other patients and medical staff, resulting in many cases of secondary infection.

As shown in the graph above, the highest number of confirmed MERS cases outside of the Middle East region was found in Korea.

The response to MERS completely exposed contradictions in the national quarantine system, as well the healthcare system. KCDC and local government entities all proved inadequate in their ability to respond to the public health crisis caused by this infectious disease. There was confusion due to lack of clarity in the delegation of roles between the central and regional governments and the cooperation system between health authorities and medical institutions did not operate smoothly either. Most medical institutions, including general hospitals, small-to-medium-sized hospitals and clinics, were not prepared to deal with healthcare-associated infections and as a result the infection continued to spread among patients and medical staff. In addition, problems in the transport and referral system for confirmed or suspected patients were discovered, while compensation for medical institutions and research and development of emerging infectious diseases became points of contention. Moreover, medical staff who participated in the isolation and treatment of MERS patients complained about job-related burden and stress.

The MERS outbreak exposed fundamental problems in the public healthcare system and vulnerabilities in the national quarantine system but the solutions to these problems have not been clearly identified to date. The MERS outbreak caused restrictions in Korean citizens’ day to day lives and significantly impacted the national economy. The socioeconomic impact of MERS has still not been accurately assessed. What is clear at this point is that the entire Korean society has become more interested in infectious diseases and that infectious diseases have become an agenda directly linked to public safety.

Moreover, people recognized that in order to respond to emerging infectious diseases it is necessary to continually assess and monitor infectious diseases that occur worldwide and establish manuals based on up-to-date knowledge through research, specialists and timely crisis analysis during the response process. In addition, the need to establish an infectious disease response network and partnerships between medical institutions and local government, as well as a central government, was also presented.

## 3. Study Design

### 3.1. Factor Categorization

The objective of this study was to inductively explore the factors that influence response based on studies related to disaster and infectious disease response. For this, a meta-analysis method called successive approximation was used [69]. Prior to inductive exploration of the factors that influence disaster response, sample articles were used to categorize these factors. The study also aimed to perform successive meta-analyses to present a model based on detailed explanation and revision of the previously established factors influencing disaster response. Accordingly, the present study used a rough model based on the categorization of the factors influencing disaster response presented in existing studies to perform meta-analyses on SARS and MERS cases.

In summary, after establishing the initial model, several rounds of meta-analyses were performed to refine the model. Accordingly, the incomplete preliminary theoretical framework, which can be viewed as the initial model, represented simplification and categorization of major factors through existing disaster response-related studies. The study aimed to conduct successive analyses based on the incomplete framework to present a refined model by revising the factors and the relationships between them. Accordingly, precedent studies previously examined in Chapter 2 were used to derive the factors influencing disaster response from a system perspective. On a review of numerous studies, it was discovered that the duties assigned to various organizations and agencies and the factors that actually influence disaster response show regularity [47,70,71]. Therefore, based on such regularity, the study aimed to categorize these factors according to timelines from a system theory perspective.

First, the studies that presented communication, coordination, cooperation and network from the process level as the mediating variables for effective disaster response included those by [3,50,55,71]. Other studies selected the process level variable as one variable among many independent variables in analyzing the influence on effective disaster response. It was commonly pointed out that resources related to disaster response influenced the outcome of disaster response through the interactions and coordination between organizations and agencies in the response process.

Kapucu [71] analyzed the influence of the system, organizational environment, tools for organizational capability and cooperation and the decision-making process of actors in the entire process on effective disaster response. The system was a variable that included organizational structure, culture and goals, while the environment included time pressure, uncertainty and complexity of the situation. Capability was a factor that involved decision-making support, communication tools, previous cooperation experience, flexibility in responding to disaster and immediate response capability, while actors included the number of stakeholders, experts, interdependence and trust. The study examined whether these independent variables influenced effective disaster response through cooperative decision-making processes, meaning open and honest exchange of opinions, shared model construction, negotiation and utilization of relevant knowledge and information. The proposed research model was used to conduct social network analysis through content analysis, in addition to in-depth case analysis on countries that were victims of terrorist attacks, including the US, Indonesia, Turkey, Spain and the UK. Moreover, a study by Lindell et al. [51] also revealed that various resources influenced disaster planning and such process had a significant influence on the efficiency of disaster response. On the basis of these studies, a framework consisting of independent variables having an influence on the effectiveness of disaster response through the disaster response processes was constructed. Even in studies that do not present process variables as mediating factors, the majority of process variables were selected as independent variables for analysis and thus it is necessary to reorganize and categorize the variables that were presented in previous studies.

**Figure 2:**
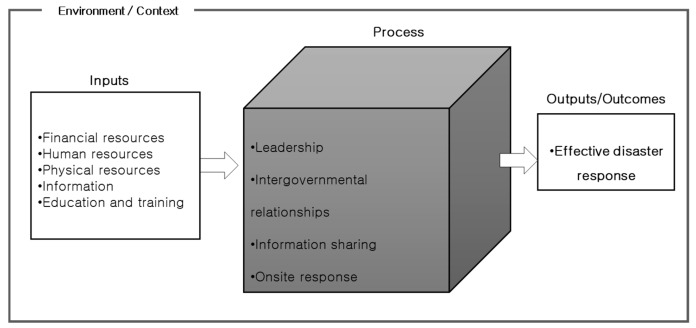
Categorization of the factors influencing disaster response.

As shown in Figure 2 the present study selected the factors influencing disaster response presented in existing studies and categorized them largely into environment, inputs and process based on system theory. This model was presented because disasters act as a single system that includes the aforementioned factors, regardless of their type (natural or social disaster) [54,72,73,74]. The strength of the influencing factors may vary depending on the type of disaster but because they were described and analyzed by the factors that are presented below, analyzing or describing social and natural disasters using different frameworks is unnecessary [57]. Based on the categorization of the influencing factors shown in the figure below, the study aimed to conduct future meta-analyses to explore detailed factors and identify the relationships between these factors.

### 3.2. Scope and Method of Analysis

For inductive exploration of the factors influencing infectious disease response, meta-analyses were performed based on the aforementioned factor categorization framework and in-depth interviews were conducted for testing and supplementation.

The present study used previous studies on disaster response to compile a list of the factors influencing disaster response and presented a theoretical framework. Moreover, the factors influencing disaster response were explored through case review, while qualitative meta-analysis was performed to identify the correlations between the factors. Qualitative meta-analysis was conducted to allow a comprehensive analysis of qualitative studies [60]. This method of analysis is different from meta-analysis which integrates results from existing empirical studies using a quantitative method [75,76]. The approach in qualitative meta-analysis involves interpretive analysis, which strives to include major concepts that appeared in individual qualitative studies but at the same time, generate a higher-level concept that can connect these concepts to a higher dimensional theoretical structure to allow for comprehensive understanding of the phenomenon and possibility of new interpretation and theoretical creation [77].

Therefore, the major goal of qualitative meta-analysis is to contribute to knowledge. From this perspective, Schreier et al. [78] listed theory building, theory explication and theory development as the three overlapping goals of qualitative meta-analysis. In the present study, the factors influencing disaster response were reviewed from existing studies in the theory building process and organized from a system theory level. Subsequently, meta-analysis was performed to explore factors through Korean studies and articles and interviews. The protocol was constructed through data collection and analysis and effort was made to ensure reliability and validity of the study [79].

Using this approach, the study was conducted systematically, from the data collection stage to the final analysis. After comprehensively collecting data, including domestic research articles, media reports and audit reports from the Board of Audit and Inspection (BAI) of Korea related to SARS and MERS cases, data to be analyzed were selected on the basis of the inclusion and exclusion criteria. The data collection method will be discussed in more detail in the data collection section.

**Figure 3:**
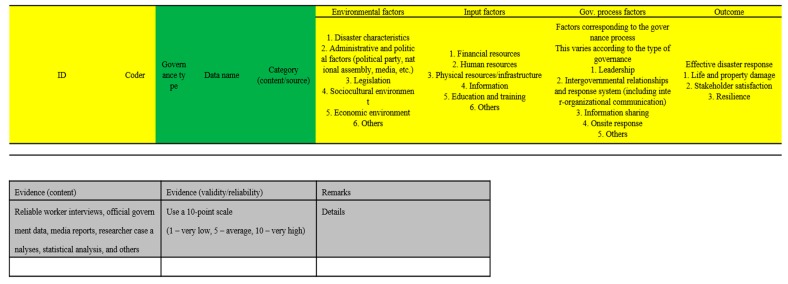
Coding framework.

The collected data were codified and categorized on the basis of the meta-analysis framework consisting of the factors influencing disaster response extracted from existing studies and theories. As shown in Figure 3, for the influencing factors identified from the data, the factor and source were recorded, and evidence of the correlation was identified. The evidence included statistical data, media reports, interviews with experts and claims made by authors. With this coding process, consistency of the results when the same analysis is performed by different researchers can be maintained and this can be used to ensure reliability.

Data for meta-analysis were collected from various sources, including listed academic journals, articles from daily and weekly periodicals and audit reports from BAI. Duplicate items were eliminated based on search results and data that met the objective of the present study were selected through in-depth reviews and discussions with fellow researchers.

Academic articles were limited to those published in journals listed in the National Research Foundation of Korea, while duplicate articles and articles with low correlation to the research question were excluded. The period of data of academic articles was from 2003 (SARS outbreak) to 2017 (at the time of the research). All searched articles were tallied and data were selected through validity testing and unanimity with fellow researchers.

Media reports were collected from daily and weekly periodicals to provide information that was not covered in academic articles. The search process used the Naver news site and the official home pages of each newspaper. The search keywords were disaster case names: SARS, MERS and different variations of these terms in Korean. Additionally, data that mentioned a disaster name along with the term “response” were reviewed. To eliminate political bias, Chosun Ilbo, Donga Ilbo, Kyunghyang Shinmun and Hankyoreh were selected from daily periodicals, while Weekly Chosun, Weekly Donga, Weekly Kyunghyang and Hankyoreh 21 were selected from weekly periodicals. The period included in data collection was set to one year to include the infection outbreak and response period between January and December 2003 for SARS and between January and December 2015 for MERS. Lastly, the homepage of BAI was searched for BAI audit reports on SARS and MERS but since audit reports for SARS did not exist only MERS cases were analyzed. Among the 59 cases that appeared as search results for MERS, the results that were unrelated to the selected cases were excluded. As a result, a total of 38 cases of audit reports for various organizations were selected for the analysis (Table 1).

## 4. Analysis of Results

The results of factors coded and explored based on aforementioned media reports, BAI audit reports, academic articles and in-depth interviews are provided here. To effectively demonstrate the results of exploring the factors influencing disaster response, analysis was performed by identifying how each factor, as an independent factor, influenced other factors; and by gathering evidence of the relationship between time, cause and outcomes.

The present study underwent the process of identifying and testing correlations through a meta-analysis of the factors influencing infectious disease response. The analysis results on the factors influencing infectious disease response were as follows.

Legislation, sociocultural factors and disaster characteristics were identified as the environmental factors influencing disaster response. With respect to SARS, although there was no legislative system for disaster response and management, some respiratory transmission diseases, including SARS, were temporarily designated as “infectious diseases subject to quarantine and surveillance” for onsite response. Enactment and amendment of laws have procedural and time requirements and thus quarantine or isolation was made possible by presenting them as subjects of quarantine and surveillance following the decree of the Minister of Health and Welfare, which actually had a positive influence on onsite response. Moreover, while legislation for MERS was in place, it was incomplete and not detailed enough. This caused confusion in the response process because of the possibility of arbitrary decisions and because it contained inaccurate information about infectious diseases, it had a negative influence on onsite response. Consequently, MERS spread to other patients, leading to a failed initial response. Moreover, unlike the SARS outbreak, when international public health crisis was declared, there was no announcement of an international public health crisis with MERS, which caused a lack of awareness on the importance of prevention and response.

Financial resources, human resources, physical resources, information and education and training were identified as the input factors influencing disaster response. Human resources also acted as a mediating factor in the relationship between legislation and the effectiveness of response to infectious diseases. In the processes of responding to SARS and MERS, problems related to human resources, especially epidemiologists, were identified. This was also very apparent in the correlations. Although epidemiological investigation in infectious disease response is very important for preventing the spread of infectious diseases and for timely response, an insufficient number of epidemiologists made it impossible to keep up with the rate at which the disease was spreading and since public health physicians were mostly responsible for epidemiological investigation, a lack of specialization was also a serious problem. Moreover, budget, the proportion of public healthcare and infection control infrastructure, such as negative-pressure units, were also found to be insufficient during both SARS and MERS outbreaks. One of the factors that was identified as being important in the correlation analysis was education and training. Since everyone may experience an actual disaster, simulated training according to given scenarios and education for response personnel are very important.

Leadership, intergovernmental relationships, information sharing and onsite response were identified as the process factors influencing response to infectious disease outbreak, while information sharing was found to influence stakeholder satisfaction. With respect to leadership, as mentioned earlier, the role of the prime minister and the president was an important factor in the implementation of timely and effective disaster response. During the SARS outbreak, Prime Minister Kun Goh was at the forefront, urging the public and departments to cooperate. On the other hand, during the process of responding to MERS, the control tower changed at least twice and the president made it clear through the spokesperson that the Blue House was not the control tower. During this process, the intergovernmental relationship was not smooth either. Moreover, poor information sharing and communication between departments and between the central and local government caused confusion and increased the level of distrust among the general public. Among the process factors, intergovernmental relationships, information sharing and onsite response were independent variables that influenced the outcome and acted as mediating factors between legislation and outcome.

Lastly, although not presented in existing analytical frameworks, the factors identified through meta-analysis and interviews were interest and cooperation from the private sector (volunteerism). With respect to interest, an analysis of SARS cases showed that the interest of local citizens, meaning regional self-centeredness, caused the designation of SARS quarantine hospitals to be nullified, acting as a factor that interfered with infectious disease response. These factors were confirmed in the interview results. The interest of the agency in charge of the control tower emerged as a factor that interfered with the infectious disease response, albeit at a different level than the interest of local citizens. The agency in charge of determining the disclosure of information was the MOHW and because the same agency was responsible for both promoting actual related projects and managing disaster, conflict of interest did not allow immediate response measures to be implemented.

Cooperation from the private sector (volunteerism) was a factor that did not appear in the meta-analysis but was identified in interviews with workers. Its correlations were not analyzed in the meta-analysis data of the present study and existing studies did not discuss the role of volunteers in infectious disease response either. However, resource support for self-quarantine patients in actual infectious disease response was lacking but active participation by volunteers played a major role in helping to slow the spread of MERS and to successfully implement self-quarantine.

Moreover, the hidden context of correlations identified through interviews, which was not identified in existing articles, was education and training. Education and training was analyzed as a factor influencing infectious disease response, while the interview results revealed that education and training not only had a direct influence on response but also had an impact on the relationship between the people in charge of disaster response. Timely response was made possible by relationships built between people in charge of disaster response through continued training, which may be attributed to the uniqueness of Korean culture.

**Figure 4:**
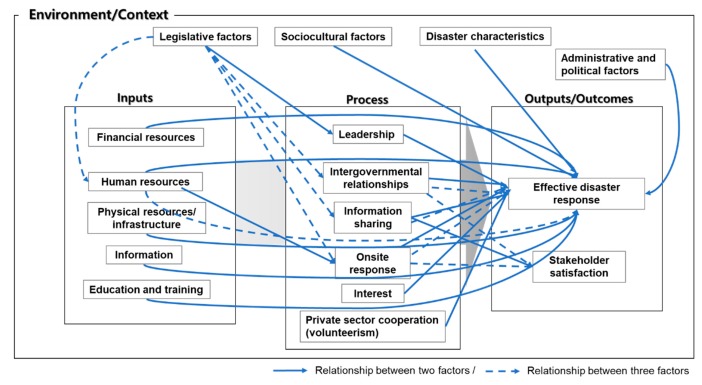
Model of factors influencing infectious disease response in Korea.

As shown earlier, the factors influencing infectious disease response in Korea were very diverse and they became more refined and detailed when compared to categorization of factors presented in the introduction of this paper. These were factors identified through meta-analyses and in-depth interviews and should be considered in the improvement of the infectious disease response system in Korea. A comprehensive model that summarizes the aforementioned exploration of the influencing factors is shown in Figure 4.

## 5. Conclusions

The present study conducted meta-analyses to comprehensively analyze the correlations of factors influencing disaster response from a Korean context. For inductive exploration of the factors influencing infectious disease response in Korea, the present study collected and selected reliable data from academic research on infectious disease response conducted in Korea, newspaper articles and audit reports from BAI. The reason for limiting the studies to those conducted within Korea was based on the determination that it was necessary to review how well domestic studies and articles explained domestic cases. The objective was to use the findings to point out the limitations of infectious disease-related studies in Korea and to present factors influencing infectious disease response within a Korean context.

The analysis results confirmed that, overall, the studies Korea focused on factors from the process aspect when analyzing the factors influencing infectious disease response. A summary of other major findings are as follows: 

First, among environmental factors, the legislative factor had direct and indirect influence on the overall process of infectious disease response. Other environmental factors were regarded as factors influencing disaster response based on their correlations but the legislative factor was considered especially important. Disaster-related legislation enacted in various forms including basic laws, manuals and code of conduct should be systematic and exhibit high integrity to allow timely and accurate response in crisis situations. However, owing to insufficiencies in many aspects, it had a negative influence throughout the entire response process.

The legislative factor indirectly influenced disaster response, making it an important factor that influences the overall disaster response process. In other words, human resource was identified as the mediating factor in the relationship between the legislative factor, human resources and onsite response. On the other hand, intergovernmental relationships, information sharing and onsite response were identified as the mediating factors in the relationships between the legislative factor, intergovernmental relationships and the effectiveness of disaster response; the relationship between the legislative factor, information sharing and the effectiveness of disaster response and the relationship between the legislative factor, onsite response and the effectiveness of disaster response, respectively. Along with the determination of mediating factors, the study also found that the establishment of legislation had an overall impact on infectious disease response.

Second, the results showed that most input factors, including physical resources, human resources and information were insufficient. Within a Korean context, it is believed that this problem stemmed from the lack of a disaster response system or many studies related to disaster response, as indicated by the fact that basic laws about disaster management were implemented in Korea from 2004. Considering that the systematization of disaster response following the passing of basic related laws was relatively recent, more detailed issues, such as securement of resources, did not draw attention until a disaster actually occurred, leading to gradual improvement. Therefore, factors related to these resources showed insufficiencies no matter which case was reviewed. However, considering the differences in the timeframes of the cases raises concern on whether the experience gained from the disaster response system is actually being used as an asset to improve the disaster response system in Korea.

Third, major findings regarding process factors were as follows. Leadership of the central government, establishment of an intergovernmental response system, the need for communication, information sharing and disclosure and onsite response were identified as key factors influencing effective infectious disease response. Existing studies have found that information sharing occurred top-down, from the central government to local government [80]. Even so, information sharing was correlated with process factors. Nondisclosure of hospital names by the government had an impact on the spread of infectious diseases and on failed initial response. Further, the general public voluntarily shared information and made the effort to share accurate information, such as creating a MERS map and sharing information on websites. In addition, the interests of local citizens and departments also acted as a factor that interfered with effective infectious disease response.

By analyzing the factors influencing infectious disease response within a Korean context, the present study presents the following theoretical and policy implications. Theoretically, the study established a model of factors influencing infectious disease response by performing inductive exploration on the factors influencing infectious disease response in Korea, which was utilized for comprehensive analysis. Policy-wise, the study aimed to emphasize the need for improvement of infectious disease response-related legislation, strengthening the authority of KCDC, which currently serves as the control tower, qualitative and quantitative supplementation of disaster response-related human resources, legal grounds for the authority of response personnel and preparation of protective measures. Lastly, since the importance of information collection and sharing and cooperation between agencies was demonstrated, it is necessary to establish a system for information sharing and disclosure, as well as a cooperation system involving the central government, the local government, health centers and medical institutions.

## Figures and Tables

**Table 1:** Analysis of targets.

Category		SARS	MERS
Academic articles	Total search results	110 cases	229 cases
	Selected for analysis	0 cases	4 cases
Media reports	Total search results	120 cases	2416 cases
	Selected for analysis	5 cases	28 cases
BAI audit reports	Total search results	0 cases	59 cases
	Selected for analysis	0 cases	38 cases
